# Essential Polyunsaturated Fatty Acids in Blood from Patients with and without Catheter-Proven Coronary Artery Disease

**DOI:** 10.3390/ijms23020766

**Published:** 2022-01-11

**Authors:** Chaoxuan Wang, Jörg Enssle, Anne Pietzner, Christoph Schmöcker, Linda Weiland, Oliver Ritter, Monique Jaensch, Ulf Elbelt, Nikolaos Pagonas, Karsten H. Weylandt

**Affiliations:** 1 Division of Medicine, Department of Gastroenterology, Metabolism and Oncology, University Hospital Ruppin-Brandenburg, Brandenburg Medical School, 16816 Neuruppin, Germany; Chaoxuan.Wang@mhb-fontane.de (C.W.); Joerg.Enssle@mhb-fontane.de (J.E.); Anne.Pietzner@mhb-fontane.de (A.P.); Christoph.Schmoecker@mhb-fontane.de (C.S.); Ulf.Elbelt@mhb-fontane.de (U.E.); 2 Medical Department, Division of Psychosomatic Medicine, Campus Benjamin Franklin, Charité—Universitätsmedizin Berlin, Corporate Member of Freie Universität Berlin and Humboldt-Universität zu Berlin, 12203 Berlin, Germany; 3 Faculty of Health Sciences, Joint Faculty of the Brandenburg University of Technology, Brandenburg Medical School and University of Potsdam, 14469 Potsdam, Germany; 4 Division of Medicine, Department of Cardiology, University Hospital Brandenburg an der Havel, Brandenburg Medical School, 14770 Brandenburg an der Havel, Germany; l.weiland@klinikum-brandenburg.de (L.W.); o.ritter@klinikum-brandenburg.de (O.R.); Monique.Jaensch@mhb-fontane.de (M.J.); n.pagonas@klinikum-brandenburg.de (N.P.)

**Keywords:** coronary artery disease, triglycerides, polyunsaturated fatty acids, n-3 PUFA, statins, arachidonic acid

## Abstract

Coronary artery disease (CAD) is the leading cause of death worldwide. Statins reduce morbidity and mortality of CAD. Intake of n-3 polyunsaturated fatty acid (n-3 PUFAs), particularly eicosapentaenoic acid (EPA), is associated with reduced morbidity and mortality in patients with CAD. Previous data indicate that a higher conversion of precursor fatty acids (FAs) to arachidonic acid (AA) is associated with increased CAD prevalence. Our study explored the FA composition in blood to assess n-3 PUFA levels from patients with and without CAD. We analyzed blood samples from 273 patients undergoing cardiac catheterization. Patients were stratified according to clinically relevant CAD (*n* = 192) and those without (*n* = 81). FA analysis in full blood was performed by gas chromatography. Indicating increased formation of AA from precursors, the ratio of dihomo-gamma-linolenic acid (DGLA) to AA, the delta-5 desaturase index (D5D index) was higher in CAD patients. CAD patients had significantly lower levels of omega-6 polyunsaturated FAs (n-6 PUFA) and n-3 PUFA, particularly EPA, in the blood. Thus, our study supports a role of increased EPA levels for cardioprotection.

## 1. Introduction

Coronary artery disease (CAD) is the leading cause of death in both developed and developing countries [1,2]. Lifestyle and environmental and genetic factors are risk factors for the development of CAD [3], with atherosclerosis being the underlying pathological mechanism.

Among other factors, triglycerides (TGs) play an important role in the development of atherosclerosis [4]. In addition, clinical trials showed that the recurrence rate of coronary events was significantly reduced if the TG level was lowered to less than 150 mg/dL in CAD patients [5].

N-3 polyunsaturated FAs (n-3 PUFA), found predominantly in fish oils, have been implicated in the prevention of CVD. Studies have shown that n-3 PUFAs have a variety of cardiovascular-protective effects, including lowering blood pressure, improving cardiac function, reducing leucocyte-derived cytokine formation, and acting as an anti-inflammatory, as well as an anti-oxidative [6]. The American Heart Association recommends that patients with documented CVD take n-3 PUFAs at a dose of approximately 1 g/day in combination with the essential FAs (EFAs) eicosapentaenoic acid (EPA, C20:5 n-3) and docosahexaenoic acid (DHA, C22:6 n-3) in the form of a fatty fish or fish oil supplement [7,8]. Similar recommendations are given by the European Society of Cardiology [9]. However, results from clinical trials are not consistent and the recently published STRENGTH trial failed to demonstrate a clinical benefit in high-risk patients treated with EPA/DHA [10], while the REDUCE-IT trial found a clear benefit in CAD patients with elevated TGs when treated with EPA [11].

Statins can significantly reduce the incidence of acute CAD in both patients with a history of CAD and patients with cardiovascular risk factors [12]. Statins inhibit the enzyme HMG-CoA reductase, which plays a central role in the production of cholesterol [13]. The lipid-lowering effect of statins has been the dominant approach to reduce and prevent CVD. As studies have confirmed that statins can lower serum cholesterol and significantly reduce CVD morbidity and mortality [14], their use is a basic principle for primary and secondary prevention of CVD. With regard to combining statins with n-3 PUFA, the JELIS study found that adding 1800 mg EPA per day to a statin medication in a group of hypercholesterolemic patients significantly decreased cardiovascular endpoints [15]. In a study exploring pitavastatin in combination with 1800 mg EPA per day for CVD treatment, it was found that patients in the combined pitavastatin/EPA treatment group had significantly higher plaque regression rates compared to pitavastatin alone [16]. In another randomized control trial (RCT) of statin use in CAD patients, compared to statins alone, patients supplemented with 3400 mg EPA and DHA per day had less fibrous coronary plaque progression [17]. Thus, the combination of statins and EPA apparently slows disease progression in CVD patients and improves outcomes, as recently confirmed by the REDUCE-IT trial [11].

Interestingly, statins can affect FA synthesis by changing Δ5-desaturase and Δ6-desaturase activities [18]. In vitro studies demonstrated that co-incubation of statins with different cell lines, such as HepG2 and THP-1 cells, significantly increased the mRNA content and protein expression of Δ5-desaturase [19,20]. This indicates that statins not only inhibit cholesterol biosynthesis, but also increase the efficiency of FA biosynthesis.

In this study, we aimed to characterize the FA composition in blood from patients undergoing cardiac catheterization for diagnosis of CAD in order to assess whether there are differences regarding essential FA, and, in particular, n-3 PUFA contents in patients with established CAD as compared to those without CAD.

## 2. Results

### 2.1. Patient Characteristics

A total of 273 patients undergoing cardiac catheterization were included in this analysis. Of those, 81 did not have signs of relevant coronary artery disease, while in 192 patients there were significant signs of CAD. Statin use was very different in both groups, with 13% in the CAD-free and 59% in the CAD group receiving statins. The patients’ general characteristics are shown in Table 1. Notably, patients in the CAD group were significantly older and heavier than those in the control group, and they had significantly lower levels of cholesterol, and HDL and LDL cholesterol, while HbA1c levels were significantly higher in the CAD group.

### 2.2. Total FAs in CAD vs. Control Patients

**Figure 1:**
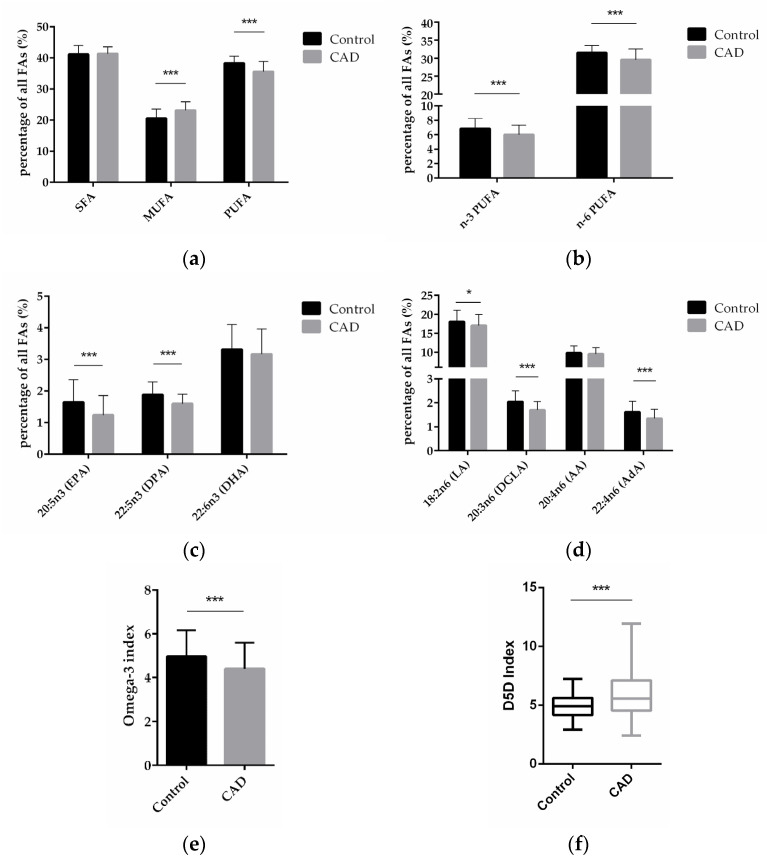
FA levels in blood from patients without and with CAD. (**a**) Relative content of SFAs, MUFAs, and PUFAs in control and CAD patients. (**b**) Relative content of n-3 and n-6 PUFAs in control and CAD groups. (**c**) Comparison of individual n-3 PUFAs in CAD and control patients. (**d**) Comparison of individual n-6 PUFAs in CAD and control patients. (**e**) n-3 index in control and CAD group. (**f**) D5D index as indicator of desaturase activity in CAD versus control patients. (*n* = 81 for the control group, and *n* = 192 for the CAD group; * indicates 0.01 < *p* < 0.05, *** indicates *p* < 0.001).

To explore the FA profiles in CAD patients to that in patients without clinically relevant CAD, total FA composition in blood from patients in both groups was analyzed and compared between the two groups. Compared with control patients, CAD patients had significantly higher levels of monounsaturated FA (MUFA, *p* < 0.0001)—comprised of palmitoleic acid (C16:1 n-7c), oleic acid (C18:1 n-9c), and nervonic acid (C24:1 n-9)—and significantly lower levels of polyunsaturated fatty acids (PUFA, *p* < 0.0001)—comprised of eicosapentaenoic acid (EPA, C20:5 n-3), docosapentaenoic acid (DPA, C22:5 n-3), docosahexaenoic acid (DHA, C22:6 n-3), linoleic acid (LA, C18: 2 n-6), dihomo-gamma-linolenic acid (DGLA, 20:3 n-6), arachidonic acid (AA, 20:4 n-6), and adrenic acid (AdA, C22:4 n-6)—but there was no significant difference in the content of saturated fatty acids (SFA)—comprised of myristic acid (C14:0), palmitic acid (C16:0), stearic acid (C18:0), arachidic acid (C20:0), behenic acid (C22:0), and lignoceric acid (C24:0)—(Figure 1a).

Compared with control patients, CAD patients had a significantly lower content of n-3 PUFAs and n-6 PUFAs (*p* < 0.0001; *p* < 0.0001) (Figure 1b). On the level of individual FAs, CAD patients had a significantly lower content of the n-3 PUFAs EPA and DPA (*p* < 0.0001; *p* < 0.0001), and DHA was also decreased in CAD patients vs. non-CAD patients, albeit non-significantly (Figure 1c). Regarding n-6 PUFAs, CAD patients had significantly lower content of LA, DGLA, and AdA (*p* = 0.0101; *p* < 0.0001; *p* < 0.0001), whereas AA did not significantly differ between the groups (Figure 1d).

The omega-3 (n-3) index is defined as the percentage of the two n-3 FAs, EPA and DHA, in total FAs. We analyzed the n-3 index in our samples presented here and found that the index was 5.0% in the control and 4.4% in the CAD patients (*p* = 0.0005) (Figure 1e).

Previous studies have indicated that increased *FADS1* gene activity could contribute to the development of CAD, as outlined in the Introduction. In order to assess *FADS1* activity on the level of the fatty acid product to substrate ratio in our study, we performed an analysis of the so-called delta-5-desaturase (D5D) index, calculated as ratio of arachidonic acid (AA, 20:4 n-6) to dihomo-gamma-linolenic acid (DGLA, 20:3 n-6). Compared with control patients, CAD patients had a significantly higher D5D index (*p* < 0.0001) (Figure 1f), indicating higher *FADS1* gene activity in CAD patients.

**Figure 2:**
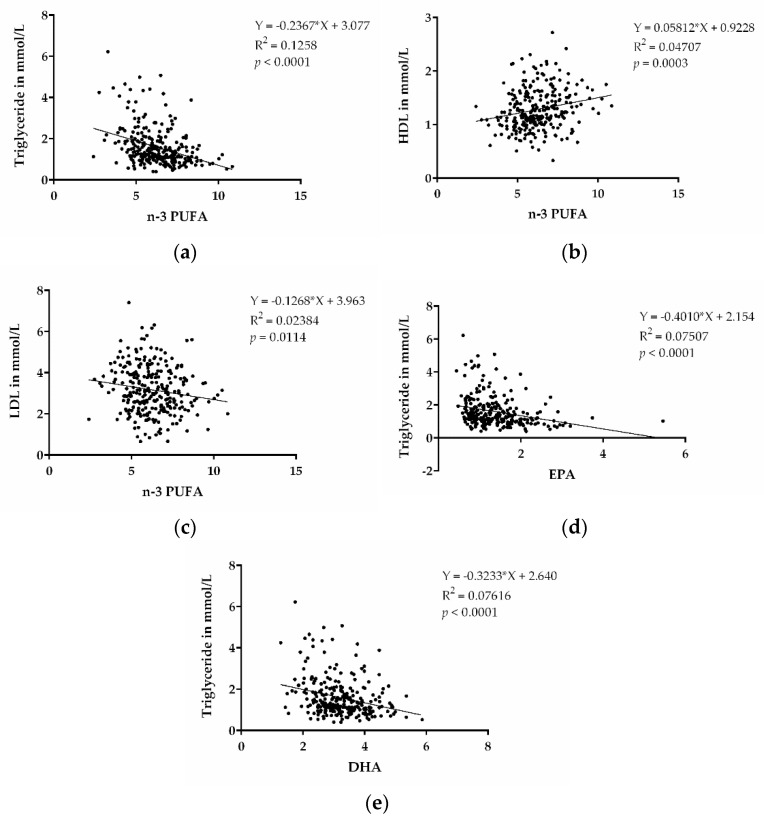
n-3 PUFAs and dyslipidemia. (**a**) Correlation between TGs and n-3 PUFA (EPA + DPA + DHA). (**b**) Correlation between HDL cholesterol and n-3 PUFA (EPA + DPA + DHA). (**c**) Correlation between LDL cholesterol and n-3 PUFA (EPA + DPA + DHA). (**d**) Correlation between TGs and EPA. (**e**) Correlation between TGs and DHA. (*n* = 273).

N-3 PUFAs were shown to decrease TGs and are approved as medical treatment for increased TG levels. In order to assess whether there were lower TG levels associated with higher n-3 PUFA levels in our cohort, we performed a correlation analysis of these parameters. TGs were inversely associated with n-3 PUFA levels (*p* < 0.0001) (Figure 2a). This indicates that high content of n-3 PUFAs, indeed, also contributes to decreased TG levels in our patient cohort. We also performed correlations between HDL and LDL with n-3 PUFA, as shown in Figure 2b,c, and found correlations for higher HDL cholesterol, as well as lower LDL cholesterol, with the n-3 PUFA levels. In addition, we also observed that EPA and DHA, individually, were inversely correlated with TG (*p* < 0.0001; *p* < 0.0001) (Figure 2d,e).

### 2.3. FAs and Statin Treatment

**Figure 3:**
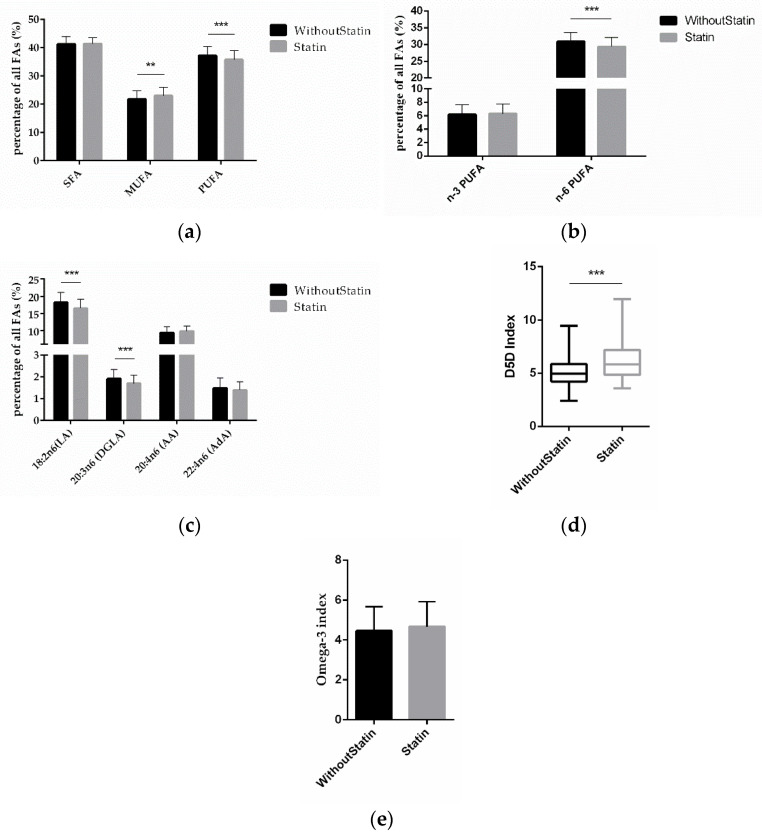
Correlation of FAs and statin treatment. (**a**) Relative content of SFAs, MUFAs, and PUFAs depending on statin treatment vs. no statin treatment. (**b**) Relative content of n-3 and n-6 PUFAs according to statin treatment. (**c**) Relative content of different n-6 PUFAs according to statin treatment. (**d**) D5D index and (**e**) omega-3 index according to statin medication. (*n* = 142 for the without-statin group, *n* = 112 for the statin group; ** indicates 0.001 < *p* < 0.01, *** indicates *p* < 0.001).

To investigate the effect of statins on FAs, we compared the FA differences between statin-treated and non-treated patients. The MUFA amount was significantly higher (*p* = 0.0011) and PUFAs were significantly lower (*p* = 0.0006) in patients on statin treatment (Figure 3a). Interestingly, it was the amount of n-6 PUFAs that was significantly lower (*p* < 0.0001) in statin-treated patients, while for n-3 PUFAs there was no significant difference according to statin treatment (Figure 3b). This was due to LA and DGLA being significantly lower (*p* < 0.0001; *p* < 0.0001), while AA and AdA were not significantly different (Figure 3c). Discernible in these data, the D5D index (*p* < 0.0001) was significantly higher in patients on statin medication (Figure 3d), while the omega-3 index remained unchanged (Figure 3e).

### 2.4. FA Comparison in Control and CAD Patients without Statin Treatment

**Figure 4:**
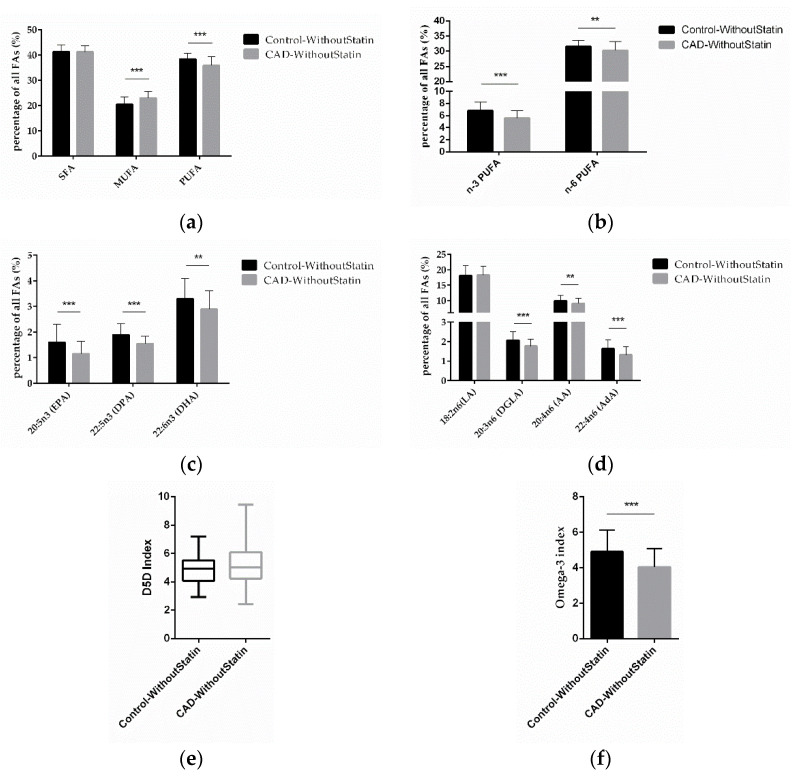
Correlation of FAs in control and CAD patient subgroups without statin treatment. (**a**) Relative content of SFAs, MUFAs, and PUFAs in control and CAD patients without statin treatment. (**b**) Relative content of n-3 and n-6 PUFAs in control and CAD patients without statin treatment. (**c**) Relative content of different n-3 PUFAs and (**d**) n-6 PUFAs in control and CAD patients without statin treatment. (**e**) D5D index and (**f**) omega-3 index in control and CAD patients without statin treatment. (*n* = 70 for the control-without-statin group, *n* = 72 for the CAD-without-statin group; ** indicates 0.001 < *p* < 0.01, *** indicates *p* < 0.001).

To determine the effect of essential FAs on CAD, we compared the differences of the FAs in the control and CAD patients, focusing on those without statin medication in both groups. We found that MUFA was significantly higher (*p* < 0.0001) and PUFA (*p* < 0.0001) was significantly lower in CAD patients without statin treatment (Figure 4a). Further analysis found that levels of n-3 PUFA and n-6 PUFA (*p* < 0.0001; *p* = 0.0029) were significantly lower in CAD patients without statin treatment (Figure 4b). The results of individual FA analysis for n-3 PUFA showed that EPA, DPA, and DHA (*p* < 0.0001; *p* < 0.0001; *p* = 0.0017) were significantly lower in CAD patients without statin treatment (Figure 4c). Regarding n-6 PUFA, DGLA, AA, and AdA (*p* < 0.0001; *p* = 0.0071; *p* < 0.0001) were also significantly lower (Figure 4d). Notably, also in these statin-free groups, a higher D5D index was found in CAD patients, although this was not significant (*p* = 0.0654) (Figure 4e), while the omega-3 index (*p* < 0.0001) was significantly lower in CAD patients (Figure 4f). After excluding the effect of statins on FAs, our results thus indicate that low levels of n-3 PUFA and n-6 PUFA, as well as higher D5D activity, might be present predominantly in CAD patients.

## 3. Discussion

In this study, we found significant differences in FAs in CAD patients as compared to those without. N-3 and n-6 PUFA content was significantly lower in CAD patients. Since n-3 PUFAs have been implicated to have cardioprotective effects, their lower content might be important in the development and progression of CAD. We further analyzed the content of different kinds of n-3 PUFA and found that EPA and DPA were significantly lower in our CAD patient cohort compared to the control cohort, with no significant difference for DHA. This is in accordance with the strong evidence for the beneficial cardiovascular effects of EPA [11,15], but less so for EPA plus DHA. For DPA, there are data from a meta-analysis based on prospective cohort studies indicating that DPA levels were negatively correlated with stroke death [21]. In a second meta-analysis based on ten prospective cohort studies with 20,460 patients, a negative association between stroke risk and circulating DPA levels, but not with EPA, had been reported [22]. In addition, we found that DHA was significantly lower in CAD patients without statin treatment. However, this difference was not observed in statin-treated patients.

Mechanistically, intestinal cells take up dietary TGs and bind to apolipoprotein (apo) B-48 to form chylomicrons, which are transported through the pre-mesenteric lymphatic vessels and then through the thoracic duct into the blood circulation, where they are rapidly hydrolyzed by lipoprotein lipase [2] along the surface of the capillary lumen, leading to an elevation of free FAs and chylomicron residues [23]. High levels of TGs increase the risk of CVD. Studies have indicated that n-3 PUFA can increase lipoprotein lipase (LPL) activity and alter the kinetics of apoB100-containing lipoproteins [24]. A further study published by Allaire et al., found that EPA and DHA independently increased the rate of VLDL-apoB100 catabolism compared to the control [25]. In addition, n-3 PUFA is a ligand of the nuclear receptor family of transcription factors PPAR, which induces hepatic β-oxidation to reduce endogenous lipid production by activating PPARα [26,27]. In our study, we found that TG levels showed an inverse trend with the n-3 PUFA, in accordance with these mechanisms and the established effect of n-3 PUFA decreasing TG levels in the blood.

The importance of the effect of n-3 PUFA on TG-lowering and risk reduction of events in CAD is strongly supported by data from the REDUCE-IT trial [11,28]. Our previous study showed increased anti-inflammatory, anti-thrombotic, and antioxidant lipid metabolites in the blood of patients with n-3 PUFA supplementation [29]. This mechanism of action may also contribute to TG-lowering, as well as anti-atherosclerotic effects that have been postulated for n-3 PUFA in the context of CVD. 

**Figure 5:**
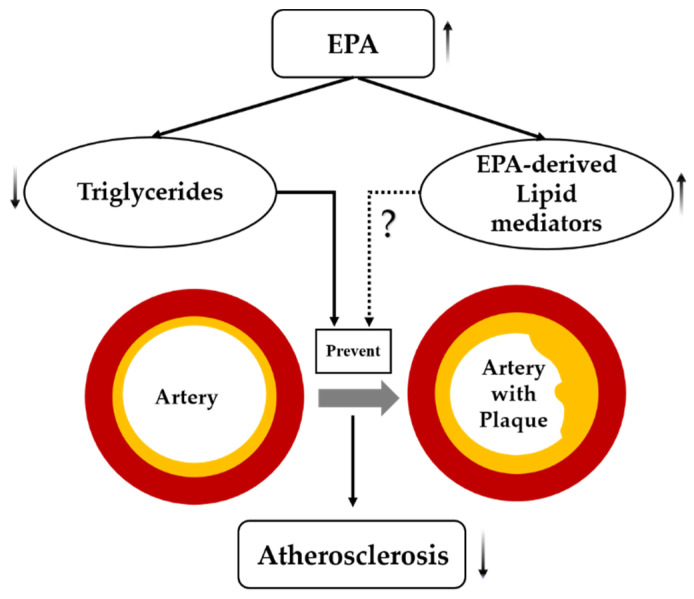
Postulated mechanism(s) by which EPA suppresses atherosclerosis. Dashed lines indicate possible effects of EPA that need further investigation, solid lines indicate effects of EPA that are well established in the scientific literature, arrows indicate increased or decreased.

EPA serves as an n-3 PUFA, playing an important role in cardiovascular diseases [30]. High purity EPA showed beneficial effects and regression of atherosclerotic plaques [31]. Growing evidence supports the use of EPA, particularly, as an anti-atherosclerotic agent [32]. Apparently, it is EPA that reduces the residual risk after statin therapy by directly affecting atherosclerotic plaques [33]. It has been postulated that EPA—besides its well-established TG-lowering effect—also acts through EPA-derived anti-inflammatory lipid mediators [34] (Figure 5).

The role of n-6 PUFAs in CVD is still inconclusive, but our study found significantly lower content in the total amount of n-6 PUFA in CAD patients. The analysis of individual n-6 PUFA content showed that the content of LA, DGLA, and AdA was significantly lower, whereas AA content was similar in CAD patients as compared to controls. Although AA can be metabolized via the cyclooxygenase and lipoxygenase pathways to produce pro-inflammatory mediators, such as prostaglandins and leukotrienes [34], which may be related to the development of CAD, our study did not show any difference in AA content between CAD and control patients.

The clinical impact of n-6 PUFA on CVD risk and mortality remains controversial [35]. Yang et al., found that n-6 PUFA concentrations were negatively associated with CVD risk, and especially high LA concentrations were significantly associated with low CVD risk and CVD mortality [36].

FADS1 is a critical enzyme for the production of long-chain PUFA, and PUFA are a precursor for many important metabolites [37]. In our study, the D5D index describing *FADS1* gene activity was higher in CAD patients compared to control patients. Such results have also been described earlier and have been linked to the effect of specific FADS1 genotypes in combination with the impact of a western diet [38,39,40]. This could indicate that, with an n-6 PUFA-high western diet as a prerequisite, higher FADS1/D5D activity leads to the production of more AA, from which pro-inflammatory lipid mediators (LMs) are synthesized [34], which in turn promote CAD/CVD. As our previous studies indicate that PUFA levels, as well as lipid-lowering treatments, such as lipid apheresis or statins, can change PUFA and lipid mediators in blood and tissue [41,42,43,44], these effects could be modified by diet and/or medications. In contrast, Yang et al., suggested that the inhibition of D5D activity accelerates the development of atherosclerosis [36].

Statins are clearly shown to lower cardiovascular risk, which is mainly due to their potent LDL cholesterol-lowering effects, and guidelines recommend statins for aggressive LDL cholesterol-lowering in patients with CVD [9,45]. Within this study we also analyzed the effect of statin treatment on FA composition in the blood. Interestingly, n-6 PUFAs, but not n-3 PUFAs, were found to be significantly lower in patients receiving statin treatment, while a previous study had described a lowering effect on both classes of FAs [46], and others had found an increase of long-chain n-6 PUFA with simvastatin [47] and lowering of FAs with an increased AA/EPA ratio [48]. Our results show a significant decrease in DGLA in patients on statin treatment, which is consistent with observations that statins increase the activity of Δ6 and Δ5-desaturase enzymes [18,49]. We also found that patients receiving statin medication had a significantly higher D5D index compared to non-statin treated patients. Given the high proportion of statin-treated patients in the CAD group (Table 1), this statin effect could explain the increased D5D ratio in the CAD patients analyzed here. However, when we analyzed the D5D index in the subgroup of patients without statin treatment, we also found a trend towards a higher D5D index in CAD patients. This is in accordance with the fact that *FADS1* (and *FADS2*) gene polymorphisms have been associated with CVD in several non-experimental studies [36,50,51,52,53].

In this context, it is a central limitation of this study that we were not able to analyze gene expression—or genotype variation—of genes involved in the elongation and desaturation of PUFAs. In particular, we did not gain any information regarding FADS1 genotype and expression levels.

Another limitation of this study is the lack of nutrition data from the patients. This, as well as analyses regarding the formation of n-3 PUFA-derived lipid mediators, remain to be addressed in future studies.

This is, thus, a pilot study establishing a difference in essential fatty acid levels between patients with and without CAD. We hope that our data will add insight and rationale for future studies to further analyze the mechanisms and clinical impacts of our findings.

The results from this study raise the question of dose- and specific statin-dependent effects on desaturase activity, as well as the role of, for example, specific FADS1 genotypes on the observed effects. We propose to study these aspects specifically in future studies. Our study therefore supports the necessity to further investigate the effect of genetic and pharmacological factors on FA synthesis in CAD and to explore how FADS1 genotypes modify the synthesis of AA from DGLA.

Most importantly, however, our study shows lower EPA levels in CAD patients, confirming the emphasis on EPA as a potentially central compound mediating the biological effects of omega-3 polyunsaturated fatty acids, and provides additional support to the concept of cardioprotection due to increased EPA levels in humans.

## 4. Materials and Methods

### 4.1. Study Population

Patients admitted for invasive coronary angiography were recruited at the University Hospital Brandenburg/Havel, Germany. Patients had either known CAD with suspicion for progress of the disease, or were referred for suspected CAD. Based on the findings of the invasive angiography, patients were then divided in the CAD group by using at least a 50% diameter stenosis as a cut off for the presence of (obstructive) CAD. Control patients were categorized as not having relevant CAD.

Exclusion criteria were active cancer disease, the inability to give informed consent, and an age of <18 years. Written informed consent was obtained from all subjects. The study was approved by the ethics committee of the medical association of the state of Brandenburg (AS69(bB)/2016).

### 4.2. Sample Collection

Blood samples were collected after overnight fasting and before the cardiac catheterization procedure. EDTA tubes were stored in −80 °C until FA analysis. A routine lipid panel (total cholesterol, LDL-C, HDL-C, and TGs) was measured by standard validated clinical assays immediately after sampling in the central laboratory of the hospital.

### 4.3. Sample Preparation

50 μL of full blood per sample was used for the gas chromatography (GC) preparation. Methylation and extraction of FAs were carried out on the basis of an established protocol [54]. Briefly, frozen samples were thawed at room temperature. All samples were then mixed with 50 µL pentadecanoic acid (PDA, 1 mg/mL, Merck Schuchardt OHG, Hohenbrunn, Germany) as internal standard, 500 µl borontrifluoride (BF_3_, Sigma-Aldrich Chemie GmbH, Taufkirchen, Germany) in 14% methanol (Merck KGaA, Darmstadt, Germany), and 500 µL *n*-hexane (Merck KGaA, Darmstadt, Germany) in glass vials and tightly closed. After vortexing, samples were incubated for 60 min in a preheated block at 100 °C. After cooling down to room temperature, the mixture was added to 750 µL water, vortexed, and extracted for 4 min. Then all samples were centrifuged for 5 min (RT, 3500 rpm). From each sample, 100 µL of the upper n-hexane layer was transferred into a micro-insert (placed in a GC glass vial), tightly closed, and analyzed by GC.

### 4.4. Determination of FAs Using GC

GC was performed on a 7890B GC System (Agilent Technologies, Santa Clara, CA, USA) with an HP88 Column (112/8867, 60 m × 0.25 mm × 0.2 µm, Agilent Technologies, Santa Clara, CA, USA), with the following temperature gradient: 50 °C to 150 °C with 20 °C/min, 150 °C to 240 °C with 6 °C/min, and 240 °C for 10 min (total run time 30 min). Nitrogen was used as carrier gas (constant flow 1 mL/min). 1 μL of each sample was injected into the injector (splitless injection, 280 °C). The flame ionization detector (FID) analysis was performed at 250 °C with the following gas flows: hydrogen 20 mL/min, air 400 mL/min, and make up 25 mL/min. Methylated FAs in the samples were identified by comparing the retention times with those of known methylated FAs of the Supelco^®^ 37 FAME MIX standard (CRM47885, Sigma Aldrich, Laramie, WY, USA). Analysis and integration of the peaks were carried out with OpenLAB CDS ChemStation Edition (Agilent Technologies, Santa Clara, CA, USA). FA values are presented as percentage (%) of total FA content. For the study, 16 FAs were included as follows: myristic acid (C14:0), palmitic acid (C16:0), stearic acid (C18:0), arachidic acid (C20:0), behenic acid (C22:0), lignoceric acid (C24:0), palmitoleic acid (C16:1 n-7c), oleic acid (C18:1 n-9c), nervonic acid (C24:1 n-9), eicosapentaenoic acid (EPA, C20:5 n-3), docosapentaenoic acid (DPA, C22:5 n-3), docosahexaenoic acid (DHA, C22:6 n-3), linoleic acid (LA, C18: 2 n-6), dihomo-gamma-linolenic acid (DGLA, 20:3 n-6), arachidonic acid (AA, 20:4 n-6), and adrenic acid (AdA, C22:4 n-6). Statistical analysis of the results was carried out by unpaired Student’s t-test and Chi-square test, where appropriate, using Prism GraphPad 5. Statistical significance was assumed when *p* < 0.05 (* 0.01 ≤ *p* < 0.05; ** 0.001 ≤ *p* < 0.01; *** *p* < 0.001).

### 4.5. N-3 Index and D5D Index

The omega-3 (n-3) index was defined as the percentage of the two n-3 FAs (FAs), EPA and DHA, of total FAs in analogy to the n-3 index level defined previously [55]. A low n-3 index has been suggested as a risk factor for CVD and its recommended range is between 4% and 8% [56].

Genes involved in the processing (elongation and desaturation) of long-chain (lc) PUFAs from short-chain (sc) precursors have been implicated in conferring diet-dependent risks of CVD [34,53,57]. The rate-limiting steps in the synthesis of lc PUFAs from sc PUFAs are catalyzed by the two FA desaturases, delta-5 desaturase (D5D) and delta-6 desaturase (D6D) [58], and encoded by FA desaturase 1 (*FADS1*) and FA desaturase 2 (*FADS2*), respectively. *FADS* gene cluster polymorphisms are, in addition to the nutritional regulation of FA supply and composition, a very important regulator of lc PUFA synthesis [59,60]. From the ratio of the FADS1 product AA to its substrate dihomo-gamma-linolenic acid (DGLA), the so-called delta-5 desaturase index (D5D index) can be calculated [61,62]. Considering this parameter, it is thus possible to draw conclusions about the efficiency of *FADS1* activity/gene expression.

## 5. Conclusions

We found lower levels of essential n-3 and n-6 PUFA in patients with catheter-proven CAD. Furthermore, there was an indication of increased desaturase activity leading to a higher D5D ratio in patients with CAD. In particular, EPA levels were significantly lower in CAD patients, supporting the concept of EPA-mediated cardioprotection.

## Figures and Tables

**Table 1:** Differences between the groups were tested with the unpaired Student’s *t*-test and Chi-square test. Data are presented as mean ± standard error of the mean.

Patient’s Characteristics	No CAD	CAD	
Male/Female	35/46	138/54	<0.0001
Age (years)	57.42 ± 1.67	67.92 ± 0.94	<0.0001
Weight (kg)	81.17 ± 1.90	86.42 ± 1.30	0.0241
BMI	27.77 ± 0.50	28.83 ± 0.38	0.0927
HbA1c (mmol/mol)	36.10 ± 0.44	44.43 ± 0.95	<0.0001
Cholesterol (mmol/L)	5.28 ± 0.12	4.67 ± 0.10	<0.0001
HDL (mmol/L)	1.50 ± 0.04	1.20 ± 0.03	<0.0001
LDL (mmol/L)	3.56 ± 0.11	3.01 ± 0.09	<0.0001
TGs (mmol/L)	1.34 ± 0.06	1.72 ± 0.08	<0.0001
Diabetes mellitus	1 (1%)	71 (37%)	<0.0001
Statin use	10 (13%)	102 (59%)	<0.0001

## Data Availability

Not applicable.
